# Knee osteoarthritis with a high grade of Kellgren–Lawrence score is associated with a worse frailty status, KNHANES 2010–2013

**DOI:** 10.1038/s41598-023-46558-2

**Published:** 2023-11-12

**Authors:** Sang Hyun Joo, Jin Woo Song, Kichul Shin, Min Jung Kim, Joongyub Lee, Yeong Wook Song

**Affiliations:** 1 Department of Internal Medicine, Daewoo General Hospital, Daewoo Medical Foundation, Geoje, Korea; 2 Department of Microbiology and Immunology, College of Medicine, Pusan National University, Yangsan, Korea; 3 Departement of Athletic Training, Gannon University, Erie, PA USA; 4 Division of Rheumatology, Biomedical Research Institute SMG-SNU Boramae Medical Center, Seoul, Korea; 5 Department of Preventive Medicine, Seoul National University, Seoul, Korea; 6 Medical Research Center, Institute of Human-Environment Interface Biology, Seoul National University, 103 Daehak-Ro, Iwha-Dong, Jongno-Gu, Seoul, Republic of Korea; 7 Song Rheumatology Clinic, 2F, HiBro BLDG, 503, Teheran-Ro, Gangnam-Gu, Seoul, Republic of Korea

**Keywords:** Musculoskeletal system, Rheumatic diseases, Data processing

## Abstract

Frailty as a syndrome of physical decline in late life is associated with adverse health outcomes. Knee osteoarthritis (KOA) could contribute to frailty conditions. The objective of this study was to evaluate the impact of KOA on frailty risk in a Korean National Health and Nutrition Examination Survey (KNHANES) cohort. In this study (N, total = 11,910, age; 64.10 years old [63.94–64.27; mean 95% CI], sex (female, %); 6,752 (56.69)), KOA patients were defined as those with knee joint pain and grade 2 Kellgren–Lawrence (K–L) or more on plain radiographic images who were 40 years old or older in Korean population data of KNHANES. The frailty index was calculated using 46 items related to co-morbidities and laboratory parameters. The impact of KOA on frailty risk was evaluated with logistic regression analyses. The prevalence of KOA patients was 35.6% [95% CI 34.7–36.46]. In polytomous logistic regression, the relative risk ratio (RRR) of KOA was significantly increased in the pre-frail group (2.76, 95% CI 2.30–3.31) and the frail group (7.28, 95% CI 5.90–8.98). RRR of frailty was significantly increased in patients with K–L grade 3 (1.36, 95% CI 1.13–1.63) and K-L grade 4 (2.19, 95% CI 1.72–2.79). Older age, higher BMI, smoking status, alcohol intake, low-income status, higher WBC count, higher platelet count, higher serum creatinine level and low estimated GFR were significantly associated with increased frailty risk. High hemoglobin and regular walking habits were associated with decreased frailty risk in KOA patients. In this large observation population- based survey cohort, KOA is linked to an increased risk of frailty syndrome. We found a significant connection between KOA and frailty syndrome. These results show that we need to think about the overall health of people with KOA and give them special care to prevent frailty syndrome.

## Introduction

Osteoarthritis (OA) is a common chronic degenerative joint disease, resulting in a diminished ability to adapt to external stressors^1–3^. These stressors could contribute to adverse outcomes including organ damage and mortality risk called frailty syndrome^4^. Frailty is defined as a biologic syndrome of decreased reserve and resistance to stressors, resulting from cumulative declines across multiple physiologic systems, and causing vulnerability to adverse outcomes^3,5^. Frailty syndrome known as Fried's frailty phenotype is classically defined as the presence of at least 3 of 5 specific health deficits: unintentional weight loss, exhaustion, low physical activity, slow walking speed, and reduced grip strength^3^. Fried’s frailty phenotype is classified into three categories based on the number of frailty indicators: robust (n = 0), prefrailty (n = 1 or 2), and frailty (n = 3)^3^. Any frailty indexes operationalize frailty by counting deficits; the more health deficits an individual has, the frailer they will be—i.e., the more susceptible to adverse health outcomes. The frailty index, calculated as a ratio of deficits present out of the total number of possible deficits, gives a continuous score from total fitness (0) to total frailty (1)^6,7^.

Several study groups reported the association between the frailty syndrome and OA patients performed by the Fried’s methods. Hip OA is associated with frailty and pre-frailty in older adults in a US cohort study^8^. Knee OA is closely related to frailty and cognitive frailty conditions in a Thailand and US cohort study^9,10^.

Recently, the prevalence and clinical features of frailty syndrome in knee OA patients have been studied in several countries. Knee pain could increase the risk of developing prefrailty and frailty^11,12^.

The prevalence of frailty and pre-frailty is known to be high in knee OA. It is associated with aging, severe knee OA symptoms, malnutrition, physical activity, and functional dependence. The prevalence of cognitive frailty is not uncommon in community-dwelling elderly^9,11,13^.

However, clinical features and frailty risks in knee OA subjects based on large-scale radiographic studies have not been well reported yet. Thus, the objective of this cross-sectional study was to evaluate the impact of KOA on frailty risk in patients with knee OA patients assessed by radiographic grades in the Korean National Health and Nutrition Examination Survey (KNHANES), a large nationwide population-based survey.

## Subjects and methods

### Study design, setting, and participants

KNHANES was designed to monitor the health and nutritional status of Koreans since 1998. It is a nationwide cross-sectional survey conducted every year. The KNHANES data were released after anonymization. The study population was not involved in the design of this study. The KNHANES is a nation-wide surveillance system to monitor the health and nutritional status of the general population of South Korea. Each year, representative samples of approximately 10,000 people are selected. Health examination, health interview, and nutritional survey are then conducted. All data are available at https://knhanes.kdca.go.kr/knhanes. The survey was conducted after receiving written informed consent from all study participants.

The study protocol was reviewed and approved by the Institutional Review Board of the Korea Disease Control and Prevention Agency (No: 2013-12EXP-03-5C, 2018–01-03-P-A). The study was performed in accordance with the Declaration of Helsinki.

From 2010 to 2013 (i.e. KNHANES IV-1 ~ KNHANES V-3), KNHANES performed the special project for the radiographic studies of knee joints in the subjects aged 40 years and older. Knee OA patients with Kellgren-Lawrence (K-L) grade (possible grade: 0 to 4) of 2 or more (based on plain knee radiographic studies) and knee joint pain were included. We excluded the patients with rheumatoid arthritis in our data processing^14^.

### Frailty index

We developed the frailty index using a cumulative deficit model, including symptoms, signs, abnormal laboratory values, disease status, and disabilities according to the Rockwood clinical frailty scale^7^. Rockwood’s frailty index was calculated as a ratio of deficits present out of the total number of possible deficits. The index gives a continuous score from total fitness (0) to total frailty (1). This means that the more deficits an individual has, the frailer they are. Our frailty index was constructed based on 46 items from the KNHANES data. These items included comorbidities, functional abilities, signs and symptoms, and laboratory values according to the Rockwood clinical frailty scale^6,7^. Comorbidities (46 items) included hypertension, pulse irregularity, myocardial infarction, angina pectoris, hypercholesterolemia, decreased high-density lipoprotein, hypertriglyceridemia, dyslipidemia, low vitamin D, diabetes, increased hemoglobin A1c, thyroid disease, dyspnea, bronchial asthma, chronic obstructive pulmonary disease, pulmonary tuberculosis, chronic hepatitis B, chronic hepatitis C, liver cirrhosis, renal failure, history of anemia, stroke, history of arthritis, limitation of motor function, chewing difficulty, weight loss, fatigue, the idea of suicide, decreased mobility, disability of self-care, disability of usual activities, pain/discomfort, anxiety/depressive mood, major depression, smoking status (those who had smoked 100 cigarettes or more in their whole life), current smoking (those who were currently smoking and had smoked 100 cigarettes or more in their whole life), stomach cancer, colon cancer, lung cancer, hepatic cell carcinoma, breast cancer, cervical cancer, and thyroid cancer. We classified subjects into robust (frailty index ≤ 0.10), pre-frail (0.10 < frailty index ≤ 0.21), and frail (frailty index > 0.21) groups^6,7^.

### Statistical analysis

**Figure 1:**
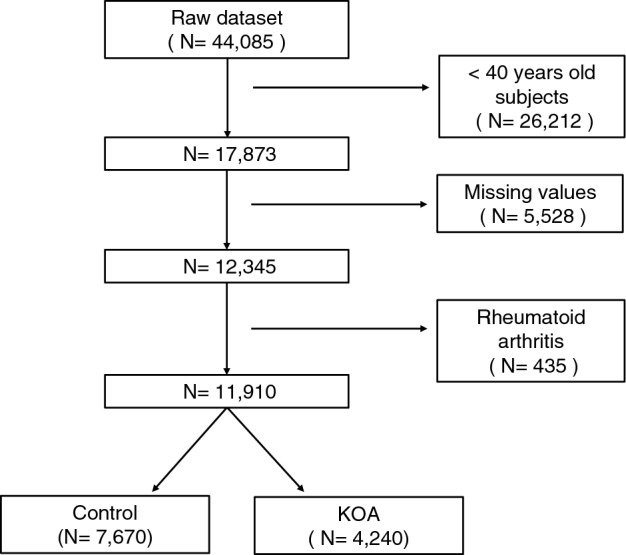
Case selection flow chart of radiographic study subjects in the Korean National Health and Nutrition Examination Survey (January 2010–December 2013) (Missing values is defined as the data value that is not stored for a variable in the observation of interest).

We analyzed the relative risk ratio (RRR; 95% CI) of frailty in control (n = 7670) and KOA subjects (n = 4240) (Fig. 1) by polytomous logistic regression. RRR was adjusted by age, sex, body mass index (BMI), smoking status, alcohol intake, low-income status, hemoglobin (Hb), hematocrit, white blood cell (WBC), platelet (PLT), blood urea nitrogen (BUN), serum creatinine, and estimated glomerular filtration rate (GFR). We analyzed clinical data in KOA subjects by analysis of covariance (ANCOVA) after adjusting for age, sex, and BMI. All statistical analyses were performed using Stata software V.16.0 (StataCorp, 4905 Lakeway Drive, College Station, Texas 77845 USA). Statistical significance was considered when the two-sided *p*-value was less than 0.05. The prevalence of KOA patients analyzed by estimated proportion (mean and 95% CI).

## Results

### Study patients

From 44,085 subjects, 17,873 subjects aged ≥ 40 years were chosen. A total of 5,528 patients were excluded due to missing values. After excluding 435 patients with rheumatoid arthritis patients, 11,910 individuals were finally evaluated in this study (Fig. 1). The prevalence of KOA patients was 12.50% [95% CI 11.93–13.10].

Baseline characteristics of study subjects according to three frailty groups are summarized in Table 1.

**Table 1:** Baseline characteristics of total study subjects (adjusted by age, sex and body mass index).

	Total (N = 11,910)	Robust (n = 4793)	Pre-frail (n = 5465)	Frail (n = 1652)	*p* value
Sex (female; %)	6752 (56.69)	2936 (43.48)	2868 (42.48)	948 (14.04)	
Age (years old), mean [95% CI]	64.10 [63.94–64.27]	61.58 [61.32–61.85]	65.03 [64.79–65.27]	68.36 [67.95–68.78]	
50 ~ < 60 (n; %)	4408 (37.01)	2400 (50.07)	1715 (31.38)	293 (17.74)	
60 ~ < 70 (n; %)	3843 (32.27)	1371 (28.60)	1914 (35.02)	558 (33.78)	
70 ~ < 80 (n; %)	2949 (24.76)	782 (16.32)	1519 (27.80)	648 (39.23)	
80 ~ < 90 (n; %)	688 (5.78)	227 (4.74)	310 (5.67)	151 (9.14)	
90 ~ < 100 (n; %)	22 (0.18)	13 (0.27)	7 (0.13)	2 (0.12)	
Body mass index (kg/m^2^), mean [95% CI]	24.02 [23.96–24.07]	23.54 [23.46–23.62]	24.23 [24.15–24.31]	24.70 [24.53–24.87]	
Underweight (n; %)	339 (2.85)	156 (3.25)	142 (2.60)	41 (2.48)	
Normal weight (n; %)	7404 (62.17)	3,256 (67.93)	3257(59.60)	891 (53.93)	
Overweight (n; %)	4167 (34.99)	1381 (28.81)	2066 (37.80)	720 (43.58)	
Obese (n; %)	0	0	0	0	
Kellgren-Lawrence score, knee joint (%)					
0	4566 (38.34)	2115 (44.13)	1.992 (36.45)	459 (27.78)	
1	2873 (24.12)	1,194 (24.91)	1,344 (24.59)	335 (20.28)	
2	1792 (15.05)	696 (14.52)	837 (15.32)	259 (15.68)	
3	1842 (15.47)	596 (12.46)	886 (16.21)	359 (21.73)	
4	837 (7.03)	191 (3.98)	406 (7.43)	240 (14.53)	
Smoking (n; %)	4476 (39.30)	1238 (27.66)	2460 (54.96)	778 (17.38)	
Current smoking (n; %)	1788 (15.70)	381 (21.31)	1026 (57.38)	381 (21.31)	
Low-income status (n; %)	2918 (24.50)	1011 (34.65)	1358 (46.54)	549 (18.81)	
Alcohol intake (n; %)	4915 (43.22)	1952 (39.72)	2406 (48.95)	557 (11.33)	
Regular walking (n; %)	4200 (36.97)	1653 (39.18)	2037 (48.50)	510 (12.14)	
Hemoglobin(g/dL), mean [95% CI]	13.95 [13.92–13.98]	13.95 [13.91–13.99]	14.04 [14.00–14.08]	13.65 [13.57–13.73]	< 0.0001
Hematocrit (%), mean [95% CI]	41.48 [41.40–41.55]	41.49[41.38–41.59]	41.71 [41.60–41.82]	40.69 [40.47–40.90]	< 0.0001
White blood cell(10^3^/μL), mean [95% CI]	6.02 [5.98–6.05]	5.68 [5.63–5.72]	6.13 [6.08–6.18]	6.56 [6.46–6.65]	< 0.0001
Platelet(10^3^/μL), mean [95% CI]	248.34 [247.20–249.49]	245.90 [244.20–247.60]	248.59 [246.90–250.28]	254.15 [250.62–257.68]	< 0.0001
Blood urea nitrogen (mg/dL), mean [95% CI]	15.79 [15.71–15.88]	15.40 [15.28–15.52]	15.80 [15.68–15.93]	16.81 [16.50–17.12]	< 0.0001
Serum creatinine (mg/dL), mean [95% CI]	0.848 [0.842–0.854]	0.799 [0.794–0.804]	0.861 [0.856–0.868]	0.938 [0.908–0.970]	< 0.0001
Estimated GFR (ml/min), mean [95% CI]	84.48 [84.21–84.76]	89.03 [88.66–89.40]	83.09 [82.71–83.47]	76.71 [75.84–77.58]	< 0.0001
EQ-5D, mean [95% CI]	0.901 [0.899–0.904]	0.976 [0.975–0.978]	0.898 [0.894–0.901]	0.715 [0.705–0.726]	< 0.0001

### Relative risk ratios of the frailty and pre-frail group compared to the robust group

**Figure 2:**
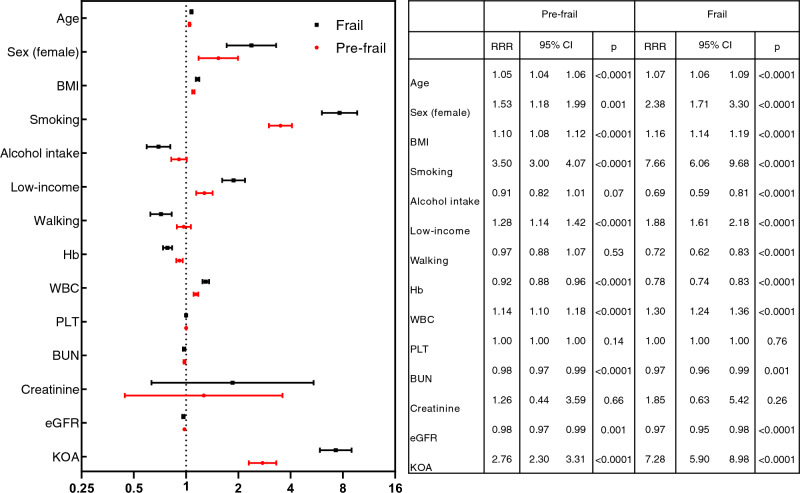
The relative risk ratio of demographic and clinical parameters in pre-frailty and frailty status compared to robust status in all radiologic study subjects (N = 11,910) in Korean National Health and Nutrition Examination Survey. *BMI* body mass index, *Hb* hemoglobin, *Hct* hematocrit, *WBC* white blood cell, *PLT* platelet, *BUN* blood urea nitrogen, *eGFR* estimated glomerular filtration rate.

RRRs were significantly increased for those with KOA (7.28 [95% CI 5.90–8.98], *p* < 0.0001), age (1.07 [95% CI 1.06–1.09], *p* < 0.0001), female gender (2.38 [95% CI 1.71–3.30], *p* < 0.0001), BMI (1.16 [95% CI 1.14–1.19], *p* < 0.0001), smoking status (7.66 [95% CI 6.06–9.68] , *p* < 0.0001), low-income status (1.88 [95% CI 1.61–2.18], *p* < 0.0001), and WBC (1.30 [95% CI 1.24–1.36] , *p* < 0.0001) in the frail group compared to the robust group. Significantly decreased RRRs were observed in those with alcohol intake (0.69 [95% CI 0.59–0.81], *p* < 0.0001), regular walking (0.72 [95% CI 0.62–0.83], *p* < 0.0001), Hb (0.78 [95% CI 0.74–0.83], *p* < 0.0001), BUN (0.97 [95% CI 0.96–0.99] , *p* = 0.001), and estimated GFR (0.97 [95% CI 0.95–0.98], *p* < 0.0001) in the frail group compared to the robust group (Fig. 2).

In the pre-frail group, significant increased RRRs were observed in those with KOA (2.76 [95% CI 2.30–3.31], *p* < 0.0001), age (1.05 [95% CI 1.04–1.06], *p* < 0.0001), female gender (1.53 [95% CI 1.18–1.99], *p* = 0.001), BMI (1.10 [95% CI 1.08–1.12], *p* < 0.0001), smoking status (3.50 [95% CI 3.00–4.07], *p* < 0.0001), low-income status (1.28 [95% CI 1.14–1.42], *p* < 0.0001), WBC (1.14 [95% CI 1.105–1.18], *p* < 0.0001). Significant decreased RRRs are observed in hemoglobin (0.92 [95% CI 0.88–0.96], *p* < 0.0001), BUN (0.98 [95% CI 0.97–0.99], *p* < 0.0001) and estimated GFR (0.98 [95% CI 0.97–0.99], *p* = 0.001) (Fig. 2).

**Figure 3:**
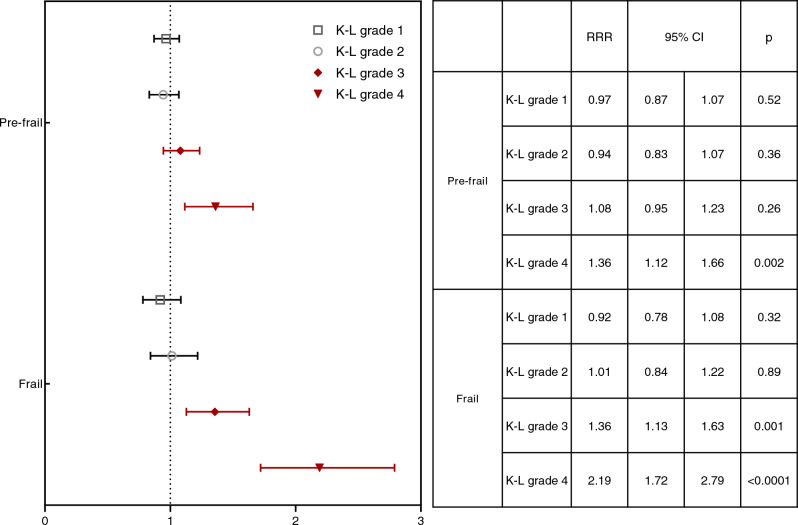
The relative risk ratio of each Kellgren–Lawrence grade of knee joint radiography in pre-frailty and frailty status compared to the robust status. *K–L grade* Kellgren–Laurence grade, *BMI* body mass index.

RRRs of frailty were significantly increased (1.36 [95% CI 1.13–1.63]) in K-L grade 3 subjects. RRRs of prefrailty and frailty were significantly increased (1.36 [95% CI 1.12–1.66] and (2.19 [95% CI 1.72–2.79] respectively) in K-L grade 4 subjects (Fig. 3) when knee radiographic studies were analyzed.

In KOA subjects according to frailty status (robust, pre-frail and frail), age (mean ± SE) (66.66 ± 0.65 years old, 69.43 ± 0.30 and 71.33 ± 0.33, *p* < 0.001), BMI (mean ± SE) (24.13 ± 0.20 kg/m^2^, 25.07 ± 0.12 kg/m^2^, and 25.46 ± 0.16 kg/m^2^, *p* < 0.0001) were increased significantly in frailty subjects (Table 2). Smoking status, current smoking, and low-income status were also significantly increased in frailty subjects (Table 2). WBC (mean ± SE) (5.50 ± 0.11(× 10^3^/μL), 5.94 ± 0.07 (× 10^3^/μL) and 6.41 ± 0.09 (× 10^3^/μL), *p* < 0.0001), PLT (mean ± SE) (246.03 ± 3.83 (× 10^3^/μL), 258.13 ± 2.39 (× 10^3^/μL) and 259.96 ± 3.35 (× 10^3^/μL), *p* = 0.04), and serum creatinine (0.73 ± 0.01 mg/dL, 0.76 ± 0.01 mg/dL and 0.86 ± 0.02 mg/dL, *p* < 0.0001) were significantly increased in those with a frailty status (robust, pre-frail and frail) (Table 2). However, Hb (13.53 ± 0.09 g/dL, 13.51 ± 0.05 g/dL and 13.27 ± 0.07 g/dL, *p* = 0.01) and estimated GFR (CKD-EPI equation) (88.54 ± 0.90 ml/min, 83.89 ± 0.51 ml/min and 76.22 ± 0.72 ml/min, *p* < 0.0001) were significantly decreased in those with a frailty status (robust, pre-frail and frail) (Table 2).

**Table 2:** Clinical features in radiologic knee osteoarthritis (KOA) and control population according to frailty status (control; n = 10,374, KOA; n = 1536) (adjusted by age, sex, and BMI).

Mean ± SE		Robust (N = 4793)	Pre-frail (N = 5465)	Frail (N = 1652)	*p*
Age (years old)	Control	61.34 ± 0.14	64.29 ± .13	66.95 ± 0.26	< 0.0001
	KOA	66.66 ± 0.65	69.43 ± 0.30	71.33 ± 0.33	< 0.0001
Sex (female; %),	Control	2748 (49.95)	2231 (40.56)	522 (9.49)	< 0.0001
	KOA	188 (15.03)	637 (50.92)	426 (34.05)	0.31
Body mass index (BMI) (kg/m^2^),	Control	23.51 ± 0.04	24.09 ± 0.04	24.34 ± 0.10	< 0.0001
	KOA	24.13 ± 0.20	25.07 ± 0.12	25.46 ± 0.16	< 0.0001
Smoking (n; %)	Control	1214 (29.14)	2317 (55.62)	635 (15.24)	< 0.0001
	KOA	24 (7.74)	143 (46.13)	143 (46.13)	< 0.0001
Current smoking (n; %)	Control	375 (22.59)	972 (58.55)	313 (18.86)	< 0.0001
	KOA	6 (4.69)	54 (41.29)	68 (53.13)	< 0.0001
Low-income status (n; %)	Control	946 (38.53)	1141 (46.48)	368 (14.99)	< 0.0001
	KOA	65 (14.04)	217 (46.87)	181 (39.09)	0.03
Alcohol intake (n; %)	Control	2604 (45.11)	2570 (44.52)	599 (10.38)	0.0003
	KOA	199 (15.24)	403 (51.60)	259 (33.16)	0.13
Regular walking (n; %)	Control	1,580 (43.66)	1,763 (47.60)	361 (9.75)	< 0.0001
	KOA	73 (14.72)	274 (55.24)	149 (30.04)	0.04
Hemoglobin, g/dL,	Control	13.97 ± 0.02	14.12 ± 0.02	13.83 ± 0.05	< 0.0001
	KOA	13.53 ± 0.09	13.51 ± 0.05	13.27 ± 0.07	0.01
White blood cell (10^3^/μL),	Control	5.69 ± 0.02	6.16 ± 0.03	6.63 ± 0.06	< 0.0001
	KOA	5.50 ± 0.11	5.94 ± 0.07	6.41 ± 0.09	< 0.0001
Platelet (10^3^/μL),	Control	245.89 ± 0.89	247.13 ± 0.92	251.40 ± 2.12	< 0.0001
	KOA	246.03 ± 3.83	258.13 ± 2.39	259.96 ± 3.35	0.04
Blood urea nitrogen (mg/dL)	Control	15.37 ± 0.06	15.78 ± 0.67	16.87 ± 0.20	< 0.0001
	KOA	16.05 ± 0.33	15.93 ± 0.17	16.68 ± 0.25	0.16
Serum creatinine (mg/dL)	Control	0.80 ± 0.00	0.88 ± 0.00	0.98 ± 0.02	< 0.0001
	KOA	0.73 ± 0.01	0.76 ± 0.01	0.86 ± 0.02	< 0.0001
CKD-EPI eGFR (ml/min)	Control	89.06 ± 0.19	82.97 ± 0.21	76.95 ± 0.56	< 0.0001
	KOA	88.54 ± 0.90	83.89 ± 0.51	76.22 ± 0.72	< 0.0001

*SE* standard error.

In addition, we studied serum iron profiles (mean ± SE) in the dataset from 2010 to 2012. It was found that serum iron was decreased in frailty subjects (robust, pre-frail and frail) with KOA (robust, prefrail, and frail) (104.05 ± 3.20 μg/dL, 104.76 ± 1.57 μg/dL and 96.57 ± 1.77 μg/dL, *p* = 0.0001). Serum ferritin was increased in frailty subjects (robust, pre-frail and frail) with KOA (79.10 ± 6.96 μg/mL, 79.86 ± 3.36 μg/mL and 86.34 ± 4.23 μg/mL, *p* < 0.0001). Moreover, serum total iron-binding capacity (TIBC) was increased in frailty subjects (robust, pre-frail and frail) with KOA (305.03 ± 3.48 μg/mL, 311.33 ± 1.89 μg/mL and 315.04 ± 2.26 μg/mL, *p* < 0.0001). These results suggest iron deficiency anemia might be related to frailty status in KOA. The regular walking habit was defined as walking for more than 30 min at a time more than five times a week. The RRR of regular walking habits revealed significant a negative correlation with frailty syndrome (0.72 [95% CI: 0.62–0.83], p < 0.0001) (Fig. 2).

## Discussion

In general, decreased BMI is a main feature of the frailty syndrome. This may lead to worsening nutritional status and a decrease in muscle mass, resulting in weight loss and muscle weakness, which are two common indicators of frailty syndrome. Increased BMI is related to the weight-bearing stress and an increased risk of knee osteoarthritis^15^. Our results revealed that increased RRR of BMI was associated with frailty in knee OA patients (Fig. 2), consistent with a previous study^16^. In our study, it can be observed that the frequency of normal weight group (Table 1) in the frailty group decreases compared to the robust and pre-frail groups, and that of the overweight group increases relatively. Also, overall distribution of the study subjects according to K-L grade, the frailty status and the BMI status show that the BMI is increased in the frailty group as the K-L grade increases. But that of the overweight group increases as the K-L grade increases (Supplement Fig. 1). These results could show the features of the survived frailty subjects in this study data.

Another previous study has reported that sarcopenic obesity is more closely associated with knee OA than nonsarcopenic obesity^13^. Sarcopenia may be the common denominator of knee OA and frailty syndrome. Sarcopenic obesity is more closely associated with knee OA than nonsarcopenic obesity, although both groups have equivalent body weights^17^. This finding supports the importance of the systemic metabolic effect of sarcopenia and sarcopenic obesity on knee OA^13,16–18^. These results suggest that different pathogenesis in knee OA can contribute to the development of frailty syndrome. We cannot analyze the sarcopenia or the sarcopenic obesity by muscle mass or body composite because the data are not available of our analysis dataset.

Kanapuru et al. have reported that lower muscle mass might be a risk factor for knee pain in patients with radiographically mild knee OA, but not in those with radiographically severe OA^19^. In women, high fat mass and low lower extremity muscle mass are associated with the presence and severity of knee OA^20^. Lower extremity muscle mass is more closely correlated with knee OA than obesity in women^20^.

Our results showed leukocytosis and thrombocytosis in KOA were accompanied by the frailty syndrome (Table 2). Furthermore, we observed an increased risk of frailty syndrome related to leukocytosis in KOA (Fig. 2). Previous studies have also reported that leukocytosis, C-reactive protein, and IL-6 levels are associated with normal aging, sarcopenia, and late-life disease (such as cardiovascular diseases)^19^. Lohman et al. have reported the importance of obesity and increased dietary inflammatory index (DII) in frailty subjects^21,22^. It has been suggested that inflammatory pathways and disordered coagulation play a role in the pathology of frailty^19^.

We hypothesized that frailty might also drive OA development by creating an inflammatory environment to interfere with normal tissue health. Molecular and biochemical changes associated with OA might in turn promote frailty, resulting in an exorable deterioration of the joint. Therefore, frailty might be considered an additional risk factor for the development of OA.

Increased serum creatinine and decreased estimated GFR (CKD-EPI equation) reflect a decreased renal function in KOA patients with frailty syndrome (Table 2). Thus, the use of renal toxic agents such as nonsteroidal anti-inflammatory drugs (NSAID) should be cautious in these patients.

Our results revealed that the frequency of regular walking had negative correlations with frailty syndromes (Fig. 2). Our results suggest the need for a long-term, adequately powered, and randomized controlled trial of exercise interventions in knee OA patients for the treatment of frailty in the elderly. Such evidence will greatly support the future design of preventive strategies against disability in older persons.

## Conclusion

In this cross-sectional population-based survey cohort, KOA is associated with an increased risk of frailty syndrome. The high K-L grade of KOA is significant the high risk of the frailty syndrome. The patients who had KOA with frailty syndrome are observed increased BMI.

### Supplementary Information

Supplementary Figure S1.

## Data Availability

The data that support the findings of this study are available from the Korean National Health and Nutrition Examination Survey (KNHANES) but restrictions apply to the availability of these data, which were used under license for the current study, and so are not publicly available. Data are however available from the corresponding authors upon reasonable request and with permission of KNHANES. The data for this study were accessed through the KNHANES homepage (https://knhanes.kdca.go.kr/knhanes/eng/index.do).

## References

[1] Salaffi F, Farah S, Di Carlo M (2020). Frailty syndrome in rheumatoid arthritis and symptomatic osteoarthritis: An emerging concept in rheumatology. Acta Biomed..

[2] Salaffi F, Di Carlo M, Carotti M, Farah S, Giovagnoni A (2020). Frailty prevalence according to the survey of health, ageing and retirement in Europe-Frailty Instrument (SHARE-FI) definition, and its variables associated, in patients with symptomatic knee osteoarthritis: Findings from a cross-sectional study. Aging Clin. Exp. Res..

[3] Fried LP (2001). Frailty in older adults: Evidence for a phenotype. J. Gerontol. A Biol. Sci. Med. Sci..

[4] O'Brien MS, McDougall JJ (2019). Age and frailty as risk factors for the development of osteoarthritis. Mech. Ageing Dev..

[5] Castell MV (2015). Osteoarthritis and frailty in elderly individuals across six European countries: Results from the European Project on OSteoArthritis (EPOSA). BMC Musculoskelet. Disord..

[6] Blodgett J, Theou O, Kirkland S, Andreou P, Rockwood K (2015). Frailty in NHANES: Comparing the frailty index and phenotype. Arch. Gerontol. Geriatr..

[7] Kang MG (2017). Association between frailty and hypertension prevalence, treatment, and control in the elderly korean population. Sci. Rep..

[8] Wise BL (2014). Frailty and hip osteoarthritis in men in the MrOS cohort. J. Gerontol. A Biol. Sci. Med. Sci..

[9] Wanaratna K, Muangpaisan W, Kuptniratsaikul V, Chalermsri C, Nuttamonwarakul A (2019). Prevalence and factors associated with frailty and cognitive frailty among community-dwelling elderly with knee osteoarthritis. J. Commun. Health.

[10] Misra D (2015). Knee osteoarthritis and frailty: Findings from the multicenter osteoarthritis study and osteoarthritis initiative. J. Gerontol. A Biol. Sci. Med. Sci..

[11] Bindawas SM, Vennu V, Stubbs B (2018). Longitudinal relationship between knee pain status and incident frailty: Data from the osteoarthritis initiative. Pain Med..

[12] Veronese N (2017). Pain increases the risk of developing frailty in older adults with osteoarthritis. Pain Med..

[13] Yoshimura N (2019). Prevalence and co-existence of locomotive syndrome, sarcopenia, and frailty: The third survey of research on osteoarthritis/osteoporosis against disability (ROAD) study. J. Bone Miner. Metab..

[14] Kellgren JH, Lawrence JS (1957). Radiological assessment of osteo-arthrosis. Ann. Rheum. Dis..

[15] Blagojevic M, Jinks C, Jeffery A, Jordan KP (2010). Risk factors for onset of osteoarthritis of the knee in older adults: A systematic review and meta-analysis. Osteoarthr. Cartil..

[16] Han CD, Yang IH, Lee WS, Park YJ, Park KK (2013). Correlation between metabolic syndrome and knee osteoarthritis: Data from the Korean National Health and Nutrition Examination Survey (KNHANES). BMC Public Health.

[17] Lee S, Kim TN, Kim SH (2012). Sarcopenic obesity is more closely associated with knee osteoarthritis than is nonsarcopenic obesity: a cross-sectional study. Arthritis Rheum..

[18] Kim YS (2012). Prevalence of sarcopenia and sarcopenic obesity in the Korean population based on the Fourth Korean National Health and Nutritional Examination Surveys. J. Gerontol. A Biol. Sci. Med. Sci..

[19] Kanapuru B, Ershler WB (2009). Inflammation, coagulation, and the pathway to frailty. Am. J. Med..

[20] Park HM (2018). Decreased muscle mass is independently associated with knee pain in female patients with radiographically mild osteoarthritis: A nationwide cross-sectional study (KNHANES 2010–2011). Clin. Rheumatol..

[21] Resciniti NV, Lohman MC, Wirth MD, Shivappa N, Hebert JR (2019). Dietary inflammatory index, pre-frailty and frailty among older US adults: Evidence from the national health and nutrition examination survey, 2007–2014. J. Nutr. Health Aging.

[22] Lohman MC, Resciniti NV, Wirth MD, Shivappa N, Hebert JR (2019). Obesity, dietary inflammation, and frailty among older adults: Evidence from the national health and nutrition examination survey. J. Nutr. Gerontol. Geriatr..

