# Supplementary material for: Knee osteoarthritis with a high grade of Kellgren–Lawrence score is associated with a worse frailty status, KNHANES 2010–2013
Source: Sci Rep. 2023 Nov 12;13:19714. doi: 10.1038/s41598-023-46558-2 (PMC10641064; doi:10.1038/s41598-023-46558-2)
Supplement: Supplementary file 1 — Supplementary Figure S1. [file 41598_2023_46558_MOESM1_ESM.pdf]

Supplementary Figure S1. Overall distribution of the study subjects according to K-L grade, the frailty status and the BMI status.

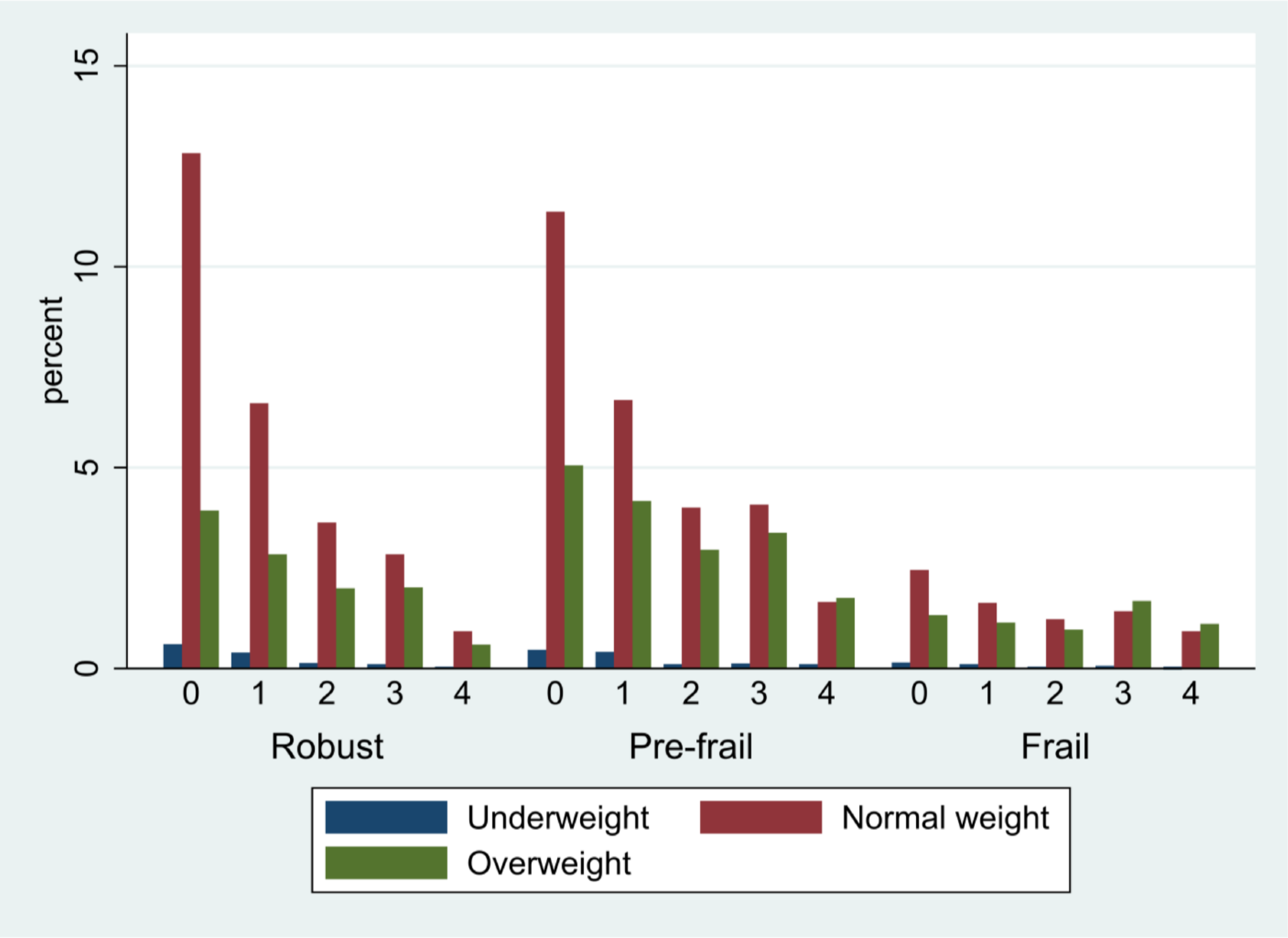

		Robust					Pre-frail					Frail				
		0	1	2	3	4	0	1	2	3	4	0	1	2	3	4
Under-weight	N	74	48	17	13	5	57	50	13	15	13	18	13	4	8	2
	%	0.6	0.39	0.14	0.11	0.04	0.46	0.41	0.11	0.12	0.11	0.15	0.11	0.03	0.07	0.02
Normal weight	N	1576	811	446	349	114	1397	821	492	501	203	301	200	150	175	114
	%	12.83	6.6	3.63	2.84	0.93	11.37	6.68	4.00	4.08	1.65	2.45	1.63	1.22	1.42	0.93
Over-weight	N	483	349	245	247	73	621	512	363	415	216	163	140	118	206	136
	%	3.93	2.84	1.99	2.01	0.59	5.05	4.17	2.95	3.38	1.76	1.33	1.14	0.96	1.68	1.11
